# Monogenic polyarteritis: the lesson of ADA2 deficiency

**DOI:** 10.1186/s12969-016-0111-7

**Published:** 2016-09-08

**Authors:** Roberta Caorsi, Federica Penco, Francesca Schena, Marco Gattorno

**Affiliations:** UO Pediatria II, Istituto G. Gaslini, Genoa, Italy

## Abstract

The deficiency of Adenosine Deaminase 2 (DADA2) is a new autoinflammatory disease characterised by an early onset vasculopathy with livedoid skin rash associated with systemic manifestations, CNS involvement and mild immunodeficiency.

This condition is secondary to autosomal recessive mutations of *CECR1* (Cat Eye Syndrome Chromosome Region 1) gene, mapped to chromosome 22q11.1, that encodes for the enzymatic protein adenosine deaminase 2 (ADA2). By now 19 different mutations in *CECR1* gene have been detected.

The pathogenetic mechanism of DADA2 is still unclear. ADA2 in a secreted protein mainly expressed by cells of the myeloid lineage; its enzymatic activity is higher in conditions of hypoxia, inflammation and oncogenesis. Moreover ADA2 is able to induce macrophages proliferation and differentiation; it’s deficiency is in fact associated with a reduction of anti-inflammatory macrophages (M2). The deficiency of ADA2 is also associated with an up-regulation of neutrophils-expressed genes and an increased secretion of pro-inflammatory cytokines. The mild immunodeficiency detected in many DADA2 patients suggests a role of this protein in the adaptive immune response; an increased mortality of B cells and a reduction in the number of memory B cells, terminally differentiated B cells and plasmacells has been described in many patients. The lack of the protein is associated with endothelium damage; however the function of this protein in the endothelial homeostasis is still unknown.

From the clinical point of view, this disease is characterized by a wide spectrum of severity. Chronic or recurrent systemic inflammation with fever, elevation of acute phase reactants and skin manifestations (mainly represented by livedo reticularis) is the typical clinical picture. While in some patients the disease is mild and skin-limited, others present a severe, even lethal, disease with multi-organ involvement; the CNS involvement is rather common with ischemic or hemorrhagic strokes. In many patients not only the clinical picture but also the histopathologic features are undistinguishable from those of systemic polyarteritis nodosa (PAN). Of note, patients with an unusual phenotype, mainly dominated by clinical manifestations suggestive for an immune-disrective condition, have been described.

Due to its rarity, the response to treatment of DADA2 is still anecdotal. While steroids can control the disease’s manifestations at high dosage, none of the common immunosuppressive drugs turned out to be effective. Biologic drugs have been used only in few patients, without a clear effectiveness; anti-TNF drugs are those associated to a better clinical response. Hematopoietic stem cells transplantation was effective in patients with a severe phenotype.

## Background

The deficiency of Adenosine Deaminase 2 (DADA2) is a recently identified disease, gathered in the family of autoinflammatory diseases, mainly characterised by early-onset polyarteritis, hemorrhagic and ischemic strokes and hypogammaglobulinemia.

In February 2014 two independent studies, one held by the American National Institutes of Health in Bethesda [1] and the other one by the Israeli Sharee Zedek Medical Center in Jerusalem [2], identified this new clinical entity, often familial, characterised by early onset livedoid rash associated with systemic inflammation (fever and elevation of acute phase reactants). Some patients presented ischemic or haemorrhagic cerebral stroke, other vasculopathy-related manifestations (hypertension, gastrointestinal symptoms), hepatosplenomegaly, peripheral neuropathy and mild immunodeficiency.

In many cases both the clinical manifestations and the histological findings were consistent with the diagnosis of polyarteritis nodosa (PAN), with childhood-onset.

The analysis of the whole exome-sequencing (WES) in unrelated affected patients identified autosomal recessive deleterious mutations in *CECR1* gene, encoding for adenosine deaminase 2 (ADA2).

The marked reduction of both plasmatic levels and enzymatic activity of ADA2 detected in affected patients respect to healthy donors [1, 2], confirmed the hypothesis that the causative mutation determines the loss-of-function of the protein. The non-affected simple-heterozygous parents displayed intermediate values of both plasmatic levels and enzymatic activity [1].

## *CECR1* gene

The *CECR1* (Cat Eye Syndrome Chromosome Region 1) gene, mapped to chromosome 22q11.1 and constituted by 10 exons [1, 2], encodes for the enzyme adenosine deaminase 2 (ADA2), a protein composed by 4 domains: the signal sequence, the dimerization domain, the putative receptor-binding domain and the catalytic domain.

**Fig. 1:**
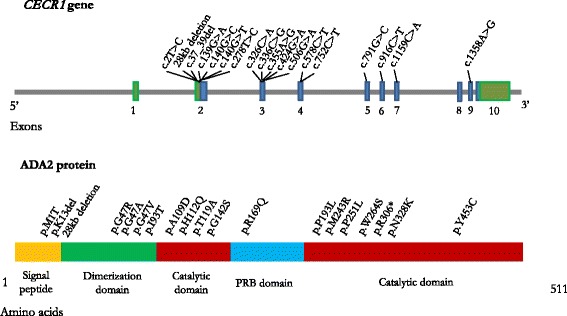
Cat Eye Syndrome Chromosome Region 1 (*CECR1*) gene and Adenosine Deaminase 2 (ADA2) protein with the mutations by now detected

The mutations detected in *CECR1* gene so far are 19, with a different prevalence according to patient’s ethnicity (Table 1, Fig. 1) [1–13]. The G47R mutation has been detected in homozygous state in all patients of Georgian Jewish and Turkish origin. Based on the results of the molecular analysis performed in 246 healthy donors of Georgian Jewish origin, the estimated frequency of this mutation in this population is 10 % [2].

**Table 1:** *CECR1* mutations so far detected

Mutation	Exon	HGVS sequence name	Aminoacid substitution	N° of patients	Enzymatic domain
M1T	2	c.2 T > C	Met1Thr	1 in compound heterozygosis	Signal peptide
K13del	2	c.37_39del	37_39del	2 in compound heterozygosis	Signal peptide (?)
28-kb-deletion	2	deletion	deletion	1 in compound heterozygosis	5′UTR (5′untranslated region)
G47R	2	c.139G > A	Gly47Arg	27 in homozygosis	Dimerization
				1 in compound heterozygosis	
G47A	2	c.140G > C	Gly47Ala	2 in compound heterozygosis	Dimerization
G47V	2	c.140G > T	Gly47Val	1 in compound heterozygosis	Dimerization
I93T	2	c.278 T > C	Ile93Thr	1 in compound heterozygosis	Dimerization
A109D	3	c.326C > A	Ala109Asp	1 in compound heterozygosis	Catalytic
H112Q	3	c.336C > G	His112Gln	1 in compound heterozygosis	Catalytic
T119A	3	c.355A > G	Thr119Ala	4 in compound heterozygosis	Catalytic
G142S	3	c.424G > A	Gly142Ser	4 in compound heterozygosis	Catalytic
R169Q	3	c.506G > A	Arg169Gln	15 in homozygosis	PBR (putative receptor-binding)
				9 in compound heterozygosis	
P193L	4	c.578C > T	Pro193Leu	1 in compound heterozygosis	Catalytic (?)
M243R	4	NA	Met243Arg	2 in compound heterozygosis	Catalytic
P251L	4	c.752C > T	Pro251Leu	4 in compound heterozygosis	Catalytic
W264S	5	c.791G > C	Trp264Ser	1 in compound heterozygosis	Catalytic
R306*	6	c.916C > T	p.Arg306*	1 in compound heterozygosis	Catalytic (?)
N328K	7	c.1159C > A	Cys1159Arg	2 in compound heterozygosis	Catalytic
Y453C	9	c.1358A > G	Tyr453Cys	3 in compound heterozygosis	Catalytic

Legend: HGVS: Human Genome Variation Society

*NA* not available

Conversely, the R169Q is the mutation more frequently detected in the European Caucasian population [1, 2, 7, 12, 13].

The mutations so far detected affect the Signal peptide (*n* = 2), the 5’ untranslated region (*n* = 1), the dimerization domain (*n* = 4), the putative receptor binding (*n* = 1) and the catalytic domain (*n* = 11) (Table 1, Fig. 1) [1–13]. Moreover two patients with a homozygous deletion on 22.11.1 chromosome (encompassing *CECR1* gene) have been recently described [14].

### ADA2 protein and pathogenetic mechanisms

The enzyme Adenosine Deaminase (ADA) plays a key role in the purine metabolism converting adenosine to inosine and 2′-deoxyadenosine to 2′-deoxyinosine [15].

The two major ADA isoforms are ADA1, whose deficiency is cause of a severe combined immunodeficiency (SCID) [16], and ADA2.

Even if the two proteins have partial structural homology, the two isoenzymes differ in many aspects: the affinity of ADA2 for molecules of adenosine and deoxy-adenosine is about 100 times lower than that of ADA1; consequently, at physiological concentrations of substrate, the deaminase activity of ADA2 is almost absent [17].

While ADA1 is monomeric and acts primarily intracellularly, ADA2 is dimeric and secreted in the extracellular environment where it exerts its main functions. For this reason ADA2 is clearly detectable in the plasma. Finally, while ADA1 is ubiquitally expressed in all cell types, ADA2 is mostly expressed by monocytes and other cells of the myeloid lineage [17].

**Fig. 2:**
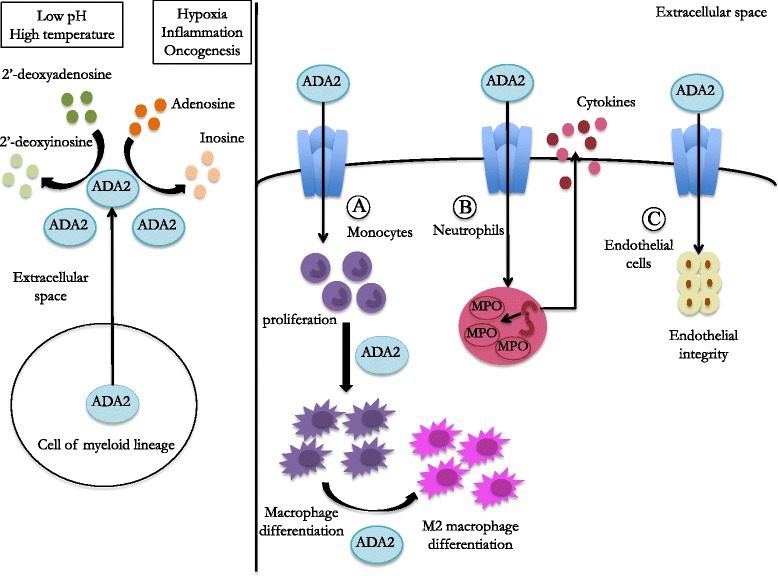
Production and physiological role of Adenosine Deaminase 2 (ADA2). ADA2 is produced and secreted by cells of myeloid lineage; it exerts its enzymatic activity in the extracellular space, especially in the presence of a low pH or high temperature. On monocytes (**a**) ADA2 acts as growth-factor with an autocrine activity: it induces monocytes’ proliferation and promote the differentiation of M2 anti-inflammatory macrophages. On neutrophils (**b**) ADA2 induces the gene of expression of some pro-inflammatory proteins, such as myeloperoxidase (MPO) and neutrophils’ activations, leading to the secretion of pro-inflammatory cytokines. There are indirect evidences of a possible role of ADA2 as growth-factor for endothelial cells (**c**)

ADA2 is more stable at high temperatures and the optimum pH for its activity is generally acid (about 6.5), which suggests a specialized role of this enzyme in conditions of hypoxia, inflammation and oncogenesis; in these conditions its deaminase activity is higher [17] (Fig. 2).

The capacity of binding receptors involved in the signal transduction of different pathways (such as proteoglycans), confers to ADA2 a growth-factor like action; for this reason ADA2 is considered an Adenosine Deaminase-related Growth-Factor (ADGF) [17–19] (Fig. 2).

ADA2 displays also an autocrine activity: the protein, released by activated monocytes, is able to induce monocyte proliferation and macrophage differentiation [20]; *CECR1* silencing in myeloid cells is in fact associated to a reduced differentiation of monocytes to macrophages [1]. This activity has been demonstrated to be mediated by the direct binding of cellular receptors, and therefore to be independent from the enzymatic activity [20] (Fig. 2).

ADA2 seems to be also involved in the balance between pro-inflammatory (M1) and anti-inflammatory (M2) monocytes; its absence has been in fact associated with a defect in differentiation of M2 macrophages, which leads to a prevalence of pro-inflammatory M1 cells [1].

Microarray analysis in two DADA2 patients showed a marked up-regulation of neutrophils-expressed genes. Intracellular staining revealed an increased expression of myeloperoxidase (MPO) in peripheral blood mononuclear cells compared to controls [8] (Fig. 2). According with these findings, the assessment of cytokine levels performed in the serum of few described patients revealed an increase of pro-inflammatory cytokines: in the two patients carrying a homozygous deletion of 22q11.1 chromosome increased levels of both IL-1β and TNFα were detected [14], while in another case the detection of IL-6 revealed increased levels [7]. These data are in contrast with the results obtained in the NIH study, in which the cytokine assay performed in the supernatants of the whole blood cell cultured with different stimuli did not reveal any significant difference between patients and healthy donors [1]. Further studies on larger series of patients are therefore needed in order to investigate the cytokines’ pattern in DADA2; in particular the cytokines’ production should be assessed in stimulated PBMCs and should take into consideration the disease activity.

It has been also postulated that the deregulation of purinergic stimulation, due to the decrease of the enzymatic activity of ADA2, may play a pro-inflammatory role. Adenosine is in fact an important signaling molecule that can modulate the inflammatory response; its concentration in tissues is normally low and increases in condition of cellular stress, ischemia or inflammation [21]. The accumulation of adenosine can influence the inflammatory response by binding several receptors that lead to inflammation, tissue damage and fibrosis [21]. However, the plasmatic levels of adenosine and deoxiadenosine in few DADA2 patients has been detected within the normal range [1, 2].

Since hypogammaglobulinemia has been described in some patients, adaptive immunity has been investigated in ADA2 patients. A reduction in the number of memory B cells, terminally differentiated B cells and plasmacells has been described [1, 7]; moreover co-culture experiments have enlightened an increased mortality of B cells [1]. Not univocal results have been detected concerning the T cells function. In fact, while in the NIH study ADA2 mutations seem not to affect T lymphocyte number and activation [1], in a more recent study an increase of regulatory T cells and a decrease of CD8+ and CD4 + memory T cells have been detected in one patient with DADA2 [7]. In addition a reduced number of Th1, Th2 and follicular T helper (Tfh) cells has been observed in the same patient.

The reason why endothelium represents the main target of inflammation in DADA2 is still largely unknown. ADA2 acts as a growth-factor for endothelial cells. In fact, even if it has been demonstrated that endothelial cells do not express *CECR1* gene, the deficiency of ADA2 is associated to a damage of vascular endothelium and to an over expression of activation markers [1]. The knockdown animal model for *CECR1* gene (zebrafish) displays cerebral haemorrhages without morphologic alteration in the vascular structure; these episodes recovered following the transfection of non-mutant human *CECR1* messenger RNA [1]. In the same way, monocytes of patients with DADA2 led to destruction of co-cultured human microvascular endothelial cells [1].

Due to the rarity of the disease, all available data on the pathogenic consequences of ADA2 defect in humans come from few patients; further studies are therefore needed in order to better enlighten the activity of ADA2 in the innate and adaptive immune response and its role in the endothelium homeostasis.

### Clinical manifestations

DADA2 can be defined as an inflammatory vasculopathy with a wide range of clinical manifestations, possibly associated with an immunodeficiency of variable severity.

**Fig. 3:**
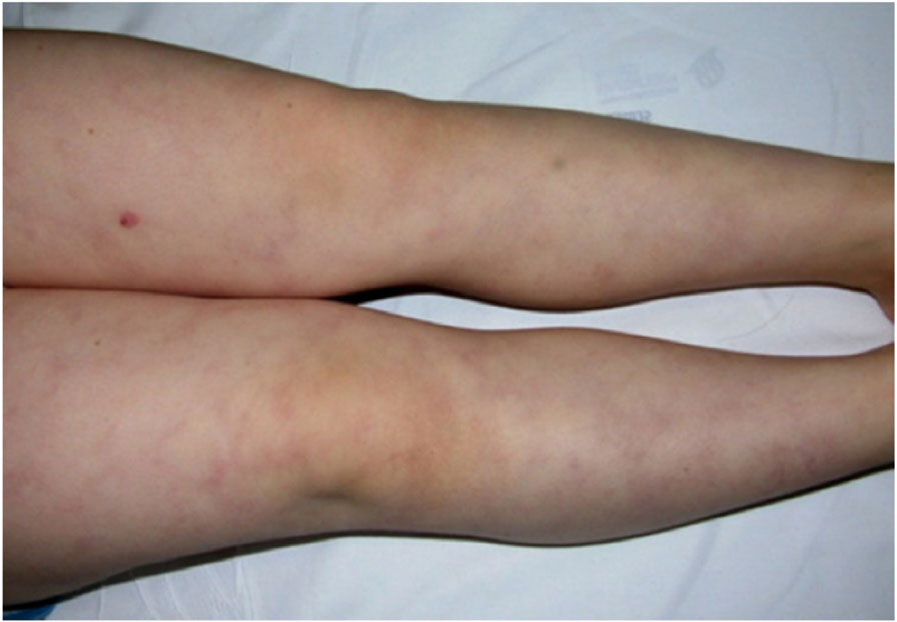
Livedo reticularis in a patient with DADA2

The disease is mainly characterized by chronic or recurrent systemic inflammation with fever and elevation of acute phase reactants, usually associated with different possible skin manifestations, ranging from the most frequent livedo reticularis (Fig. 3) to maculopapular rash, nodules, purpura, erythema nodosum, Raynaud’s phenomenon, ulcerative lesions, digital necrosis [1, 2].

The clinical picture is wide, ranging form a mild disease with a late onset skin-limited involvement to a very severe systemic phenotype (even fatal) with an early onset and a multi-organ involvement (Tables 2 and 3).

**Table 2:** Clinical manifestation of DADA2 patients so far described

Report		*CECR1* Mutation	Ethnicity	Age at onset	Fever	Skin	CNS/PNS	Gastro-intestinal	Immune/Hematologic system	ANA	ANCA	Hypertension	Other
Navon et al.	1	G47R/G47R	Georgian	2 m	Yes	Ulcerations at extremities	No	Intestinal necrosis	No	neg	neg	Yes	Coronary aneurysms
	2	G47R/G47R	Georgian	5 y	No	Livedo reticularis, nodules, purpuric rash	No	Intestinal vasculitis	No	neg	neg	No	No
	3	G47R/G47R	Georgian	7 m	Yes	Livedo reticularis, papulo-nodular rash, Raynaud’s phenomenon	Ischemic stroke, peripheral paresis of cranial nerves	No	No	neg	neg	Yes	Arthritis
	4	G47R/G47R	Georgian	3,5 y	Yes	Livedo reticularis, nodules, purpuric rash, erythema nodosum	Cranial nerve paralysis	No	No	pos	ND	No	No
	5	G47R/G47R	Georgian	2,5 y	No	Maculopapular rash, nodules	Neurosensorial hearing loss	No	No	neg	neg	No	Arthritis
	6	G47R/G47R	Georgian	2 m (died at 9 m)	Yes	Digital necrosis	Ventricular haemorrhage	Aneurism of celiac artery	No	neg	neg	No	No
	7	G47R/G47R	Georgian	2 m	Yes	Livedo reticularis, Raynaud’s phenomenon	No	Abdominal pain	No	neg	ND	No	No
	8	G47R/G47R	Georgian	6,5 y	Yes	Livedo reticularis, nodules, Raynaud’s phenomenon	No	Abdominal pain	No	neg	neg	No	No
	9	G47R/G47R	Georgian	9 y	No	Livedo reticularis	No	No	No	neg	neg	No	No
	10	G47R/G47R	Georgian	10 y	No	Livedo reticularis, nodules, Raynaud’s phenomenon, leg ulcers	No	No	No	neg	neg	No	No
	11	G47R/G47R	Georgian	59 y	No	Leg ulcers	No	No	No	neg	ND	No	No
	12	G47R/G47R	Georgian	childhood (died at 30 y)	No	Livedo reticularis, nodules, Raynaud’s phenomenon, leg ulcers with amputation	No	No	No	ND	ND	No	No
	13	G47R/G47R	Georgian	1 y	No	Livedo reticularis, nodules, Raynaud’s phenomenon	Sensitive polyneuropathy	No	No	neg	neg	No	No
	14	G47R/G47R	Georgian	4 y	Yes	Livedo reticularis, purpuric rash, skin nodules.	No	No	No	pos	ND	No	No
	15	G47R/G47R	Georgian	1 y	Yes	Livedo reticularis	No	Abdominal pain	No	neg	neg	Yes	Mesenteric and renal infarcts
	16	G47R/G47R	Georgian	18 y	No	No	No	No	No	neg	neg	No	No
	17	G47R/G47R	Georgian	28 y	Yes	Raynaud’s phenomenon, purpuric rash, leg ulcers with digital amputation	Polyneuropathy	No	No	neg	neg	Yes	Panniculitis
	18	G47R/G47R	Georgian	2 y	No	Skin nodules	Polyneuropathy	No	No	neg	neg	No	No
	19	G47R/G47R	Georgian	16 y	No	Raynaud’s phenomenon, ulceration of extremities	No	No	No	pos	neg	Yes	No
	20	R169Q/P251L	European Caucasian	1 y	Yes	Livedo reticularis	Ischemic stroke, cranial nerves (II,VI,VII) paralysis	No	No	neg	ND	Yes	No
	21	R169Q/P251L	European Caucasian	12 y	No	No	Ischemic stroke, VII cranial nerve paralysis	No	No	pos	neg	No	No
	22	R169Q/P251L	European Caucasian	1 y	No	Livedo reticularis, skin rash at extremities	Ischemic stroke VI cranial nerve paralysis, neurosensorial hearing loss	No	No	neg	neg	No	No
	23	R169Q/P251L	European Caucasian	3 m	No	Livedo reticularis, vasculitic exanthema of lower extremities	VII cranial nerve paralysis	No	No	ND	ND	No	Epididymitis
	24	G47V/W264S	Turkish	10 y	Yes	Livedo reticularis	No	No	No	neg	ND	Yes	Myalgia, abdominal and renal aneurysm
Zhou et al.	1	A109D/Y453C	European Caucasian	2 y	Yes	Livedo reticularis	Ischemic stroke	Splenomegaly	No	pos	neg	No	No
	2	G47A/Y453C	European Caucasian	1 y	Yes	Livedo reticularis, erythematous and urticarial rash	Ischemic and haemorrhagic strokes	Hepatomegaly, splenomegaly. Portal hypertension.	Pancytopenia, hypoIg	pos	neg	No	No
	3	R169Q/deletion	European Caucasian	1,5 y	Yes	Livedo reticularis, urticarial rash	Ischemic and haemorrhagic strokes	Hepatomegaly, splenomegaly, chronic gastritis	Leucopoenia, hypoIg	neg	neg	No	No
	4	G47A/H112Q	European Caucasian	5 m	Yes	Livedo reticularis, urticarial rash	Ischemic and haemorrhagic strokes	Hepatomegaly, splenomegaly, portal hypertension.	Pancytopenia, HypoIg	neg	neg	No	No
	5	R169Q/Y453C	European Caucasian	1,5 y	Yes	Livedo reticularis	Ischemic stroke	Hepatomegaly, splenomegaly.	Leucopoenia, hypoIg	neg	neg	No	No
	6	M1T/I93T	European Caucasian	1,5 y (died 16 y)	Yes	Non-Langerhans cell histiocytosis	Ischemic stroke	Hepatomegaly, splenomegaly. portal hypertension.	No	neg	neg	Yes	Evans syndrome
	7	G47R/G47R	Turkish	3,5 y	Yes	Livedo reticularis, erythema nodosum	Ischemic stroke III cranial nerve paralysis	No	No	neg	neg	No	No
	8	G47R/G47R	Tukish	4 y	Yes	Livedo reticularis, erythema nodosum	Ischemic stroke	Bowel perforation	No	neg	neg	No	Macrophage activation syndrome
	9	G47R/G47R	Turkish	9 y (died 22 y)	Yes	Livedo reticularis, ulceration of extremities	No	Hepatomegaly, splenomegaly	Leucopoenia	pos	neg	No	Renal amyloidosis, necrotising pneumonia
van Montfrans et al.	1	R169Q/R169Q	NA	6 m	No	Livedo reticularis	No	Hepatomegaly, splenomegaly	Granulocytopenia, red-cell aplasia	NA	NA	No	No
	2	R169Q/R169Q	NA	6 y	Yes	Livedo reticularis	Stroke	Hepatomegaly, splenomegaly	HypoIg, lymphopenia	NA	NA	No	No
Bras et al.	1	T119A/G142S	European Caucasian	>10 y	Yes	Livedo reticularis, ulcerations of extremities	Stroke	No	No	NA	NA	No	No
	2	T119A/G142S	European Caucasian	>10 y	Yes	Livedo reticularis, ulcerations of extremities	Stroke	No	No	NA	NA	No	No
	3	T119A/G142S	European Caucasian	>10 y	Yes	Livedo reticularis, ulcerations of extremities	Stroke	No	No	NA	NA	No	No
	4	T119A/G142S	European Caucasian	>10 y	Yes	Livedo reticularis, ulcerations of extremities	Stroke	No	No	NA	NA	No	No
Van Eyck et al.	1	G47R/G47R	NA	5 y	Yes	No	No	Splenomegaly	Lymphadenopathy, anaemia, thrombocytosis	NA	NA	No	
Garg et al.	1	G47R/R306*	Turkish	1,5 y (died 5 y)	Yes	Skin rash	Hemorrhagic and ischemic stroke. intraparenchymal haemorrhage	No	No	neg	neg	No	Acute respiratory distress syndrome
Van Eyck et al.	1	R169Q/R169Q	African/ Caucasian	6 m	No	No	Haemorrhagic stroke	Splenomegaly	Pancytopenia, hypoIg, lymphadenopathy, recurrent viral infections	NA	NA	No	No
	2	R169Q/R169Q	African/ Caucasian	5 m	No	No	TIA	Bowel perforation (ulcerative bowel disease, no signs of vasculitis)	Leucopoenia, hypoIg, lymphadenopathy, recurrent viral infections	NA	NA	No	No
Belot et al.	1	R169Q/P193L	European Caucasian	First year	Yes	Livedo reticularis, ulceration of extremities	TIA, ischemic stroke, sensitive polyneuropathy, intracerebral haemorrhage.	Bowel stenosis	HypoIg	neg	neg	No	Oral aphtae
	2	G47R/G47R	Asian	1 m	Yes	Erythema nodosum, vasculitic rash	Intracerebral haemorrhage, Ischemic stroke, Optic neuritis	No	No	NA	NA	Yes	Dactylitis, aneurysm of abdominal vessels
Westendorp et al.	1	R169Q/R169Q	European Caucasian	3 y	No	Livedo reticularis, nodules	Ischemic stroke, peripheral neuropathy neurosensorial hearing loss	No	No	NA	NA	No	No
	2	R169Q/R169Q	European Caucasian	2 y	No	Livedo reticularis	Ischemic stroke	No	No	NA	NA	No	Autism
Gonzales Santiago et al.	1	K13del/N328K	European Caucasian	2 y	Yes	Livedo racemosa	No	No	HypoIg	NA	neg	No	No
	2	K13del/N328K	European Caucasian	5 y	No	Nodules, erytema of lower extremities	No	No	HypoIg, recurrent infections	NA	NA	No	No
Batu et al.	1	G47R/G47R	Turkish	6,5 y	Yes	Livedo reticularis,	No	Abdominal pain	No	NA	NA	No	No
	2	G47R/G47R	Turkish	4 y	Yes	Livedo reticularis, erythema nodosum, necrotic ulcers	Stroke	Abdominal pain, hypertransaminasemia	No	pos	NA	Yes	Glomerulosclerosis
	3	G47R/G47R	Turkish	10 y	Yes	Livedo reticularis	No	Abdominal pain	No	NA	NA	No	No
Van Montfrans et al.	1	R169Q/R169Q	European Caucasian	1 y	Yes	Livedo reticularis, erythema nodosum, eczema, Raynaud phenomenon	Ischemic stroke, III cranial nerve paralysis	Abdominal pain, hepatomegaly	Adenopathy, hypoIg, pancytopenia	neg	neg	No	Oral aphtae, arthralgia GH deficiency
	2	R169Q/R169Q	European Caucasian	Birth	No	Livedo reticularis, eczema	No	Abdominal pain, hepatomegaly, splenomegaly	HypoIg, anaemia	ND	ND	No	Jugular vein thrombosis, GH deficiency
	3	R169Q/R169Q	European Caucasian	6 y	No	Livedo reticularis, ulceration of extremities and trunk	No	Hepatomegaly, splenomegaly	Adenopathy, anaemia	neg	neg	No	No
	4	R169Q/R169Q	European Caucasian	3 y	No	Livedo racemosa, erythema nodosum, Raynaud phenomenon	Ischemic stroke, VI cranial nerve paralysis	Hepatomegaly, splenomegaly	HypoIg, recurrent infections, anaemia	neg	neg	No	No
	5	R169Q/R169Q	European Caucasian	1y	No	Eczema	Ischemic stroke, IV cranial nerve paralysis	Splenomegaly	HypoIg, anaemia, leucopoenia	neg	ND	No	Autism
	6	R169Q/R169Q	European Caucasian	9 m	Yes	Eczema	Ischemic stroke, IV cranial nerve paralysis	Abdominal pain, hepatomegaly, splenomegaly	HypoIg, pancytopenia	pos	pos	Yes	Oral aphtae, arthralgia
	7	R169Q/R169Q	European Caucasian	8 y	Yes	Rash	Ischemic stroke, III cranial nerve paralysis	Abdominal pain, acute liver failure	HypoIg, recurrent infections, anaemia,lymphopenia	neg	neg	No	Oral aphtae, arthralgia
	8	R169Q/R169Q	African/Caucasian	6 m	No	Eczema	Intracranial haemorrhage	Hepatomegaly, splenomegaly	HypoIg, adenopath, thrombocytopenia, leucopoenia	neg	neg	No	No
	9	R169Q/R169Q	African/Caucasian	5 m	No	No	No	Bowel ulcerations, colitis, hepatomegaly, splenomegaly	HypoIg, recurrent infections, adenopathy	neg	neg	No	No
Fellmann et al.	1	Homozygous 22q11.1 deletion (*IL17RA* and *CECR1*)	Asian	Birth (Died 16 y)	No	Ichthyosiform rash, oro-vaginal ulcerations	No	No	Recurrent infection (C. Albicans, S. aureus), neutropenia	neg	neg	No	Chronic systemic inflammation, failure to thrive
	2	Homozygous 22q11.1 deletion (*IL17RA* and *CECR1*)	Asian	2 y	No	Ichthyosiform rash	No	Splenomegaly	Oro-vaginal candidiasis, Staphylococcal skin infections	neg	neg	No	Retinal vasculitis
Schepp et al.	1	R169Q/ M243R	NA	18 y	No	No	No	Splenomegaly	HypoIg, recurrent respiratory infections	NA	NA	No	Arthralgia
	2	R169Q/M243R	NA	2 m (Died 17 y)	Yes	Erythema nodosum	Intracranial haemorrhage	Splenomegaly	HypoIg, lymphopenia, recurrent urinary tract and respiratory infections	neg	NA	No	Arthritis Failure to thrive.

Legend: *CNS* central nervous system

*PNS* peripheral nervous system

*TIA* transitory ischemic attack

*GH* growth hormone

*HypoIg* Hypogammaglobulinemia

*ND* not done

*NA* not available

**Table 3:** genotype/phenotype correlation in DADA2

Mutation	Exon	N° of patients	Associated symptoms (% of patients)
M1T	2	1 in heterozygosis	Fever Skin: Non-Langerhans cell histiocytosis CNS/PNS: ischemic stroke Visceral: involvement: hepatomegaly, splenomegaly. portal hypertension, hypertension (100 %)
K13del	2	2 in heterozygosis	Fever (50 %) Skin: Livedo racemosa (50 %), skin nodules (50 %) Immune/hematologic system: hypogammaglobulinemia (100 %), recurrent infections (50 %)
28-kb-deletion	2	1 in heterozygosis	Fever Skin: Livedo reticularis, urticarial rash CNS/PNS: ischemic and haemorrhagic strokes Visceral involvement: hepatomegaly, splenomegaly. chronic gastritis Immune/hematologic system: leukopenia, hypogammaglobulinemia (100 %)
G47R	2	27 in homozygosis	Fever (64 %) Skin: Livedo reticularis (60 %), skin nodules (35 %), ulcerations at extremities/digital necrosis (32 %), Raynaud’s phenomenon (28 %), purpuric/vasculitic rash (18 %), erythema nodosum (18 %), panniculitis (3 %) CNS/PNS: ischemic stroke (21 %), hemorrhagic stroke (7 %), intracranial haemorrhage (11 %), cranial nerve paralysis (14 %), polineuropathy (11 %), neurosensorial hearing loss (3 %). Visceral involvement: abdominal pain (21 %), intestinal vasculitis (14 %), hepatomegaly (3 %), splenomegaly (7 %), hypertransaminasemia (3 %), hypertension (25 %). Immune/hematologic system: Leucopoenia (3 %), anaemia (3 %), lymphadenopathy (3 %).
		1 in heterozygosis	
G47A	2	2 in heterozygosis	Fever (100 %) Skin: Livedo reticularis (100 %), urticarial rash (100 %) CNS/PNS: ischemic and haemorrhagic strokes (100 %) Visceral involvement: hepatomegaly (100 %), splenomegaly (100 %), portal hypertension (100 %) Immune/hematologic system: Pancytopenia (100 %), hypogammaglobulinemia (100 %).
G47V	2	1 in heterozygosis	Fever Skin: Livedo reticularis Visceral involvement: hypertension, abdominal and renal aneurysm (100 %).
I93T	2	1 in heterozygosis	Fever Skin: Non-Langerhans cell histiocytosis CNS/PNS: ischemic stroke Visceral: involvement: hepatomegaly, splenomegaly. portal hypertension, hypertension (100 %)
A109D	3	1 in heterozygosis	Fever Skin: Livedo reticularis CNS/PNS: ischemic stroke Visceral involvement: splenomegaly (100 %).
H112Q	3	1 in heterozygosis	Fever Skin: Livedo reticularis, urticarial rash CNS/PNS: ischemic and haemorrhagic strokes Visceral involvement: hepatomegaly, splenomegaly, portal hypertension Immune/hematologic system: Pancytopenia, hypogammaglobulinemia (100 %).
T119A	3	4 in heterozygosis	Fever (100 %) Skin: Livedo reticularis, ulceration of extremities (100 %) CNS/PNS: ischemic and haemorrhagic strokes (100 %)
G142S	3	4 in heterozygosis	Fever (100 %) Skin: Livedo reticularis, ulceration of extremities (100 %) CNS/PNS: ischemic and haemorrhagic strokes (100 %)
R169Q	3	15 in homozygosis	Fever (37 %) Skin: Livedo reticularis (58 %), skin nodules (4 %), ulcerations at extremities/digital necrosis (8 %), Raynaud’s phenomenon (8 %), purpuric/vasculitic rash (13 %), erythema nodosum (13 %), eczema (20 %) CNS/PNS: ischemic stroke (54 %), hemorrhagic stroke (13 %), intracranial haemorrhage (13 %), cranial nerve paralysis (37 %), polineuropathy (8 %), neurosensorial hearing loss (8 %). Visceral involvement: abdominal pain (17 %), bowel ulcerations (8 %), chronic gastritis (4 %), bowel stenosis (4 %), colitis (4 %), hepatomegaly (45 %), splenomegaly (58 %), acute liver failure (4 %), hypertension (8 %). Immune/hematologic system: hypogammaglobulinemia (62 %), pancytopenia (8 %), leucopoenia (20 %), lymphopenia (13 %), granulocytopenia (4 %), anaemia (25 %), thrombocytopenia (4 %), lymphadenopathy (25 %), recurrent infections (29 %).
		9 in heterozygosis	
P193L	4	1 in heterozygosis	Fever Skin: Livedo reticularis, ulcerations at extremities CNS/PNS: ischemic stroke, TIA, intracranial haemorrhage, polineuropathy Visceral involvement: bowel stenosis, oral aphtae Immune/hematologic system: hypogammaglobulinemia (100 %).
M243R	4	2 in heterozygosis	Fever (50 %) Skin: erythema nodosum (50 %) CNS/PNS: intracranial haemorrhage (50 %) Visceral involvement: splenomegaly (100 %) Immune/hematologic system: hypogammaglobulinemia (100 %), recurrent infections (100 %).
P251L	4	4 in heterozygosis	Fever (25 %) Skin: Livedo reticularis (75 %), vasculitic rash (50 %) CNS/PNS: ischemic stroke (75 %), cranial nerve paralysis (100 %), neurosensorial hearing loss (25 %). Visceral involvement: hypertension (25 %), epididymitis (25 %)
W264S	5	1 in heterozygosis	Fever Skin: Livedo reticularis Visceral involvement: hypertension, abdominal and renal aneurysm (100 %).
R306*	6	1 in heterozygosis	Fever Skin: rash CNS/PNS: ischemic stroke, hemorrhagic stroke, intracranial haemorrhage (100 %)
N328K	7	2 in heterozygosis	Fever (50 %) Skin: Livedo racemosa (50 %), skin nodules (50 %) Immune/hematologic system: hypogammaglobulinemia (100 %), recurrent infections (50 %)
Y453C	9	3 in heterozygosis	Fever (100 %) Skin: Livedo reticularis (100 %), urticarial rash (33 %) CNS/PNS: ischemic stroke (100 %), haemorrhagic strokes (33 %) Visceral involvement: hepatomegaly (66 %), splenomegaly (100 %), portal hypertension (33 %) Immune/hematologic system: Pancytopenia (66 %), hypogammaglobulinemia (66 %).

In most patients, a neurological involvement, affecting both the peripheral and central nervous system, has been described.

The severity of the CNS involvement is rather variable. In some patients a transitorily ischemic attack (TIA) has been described (with negative cerebral CT and/or MRI), while others developed an ischemic or hemorrhagic stroke (in few cases a ventricular haemorrhage has also been detected). Typically, the strokes associated to DADA2 are lacunar with a wide range of clinical manifestations ranging from clinically silent episodes in few cases, to severe ones leading to a permanent disability [1, 2, 10, 12].

The neuropathy ranges from a transient mononeuritis (such as a cranial nerve transient paralysis) to a permanent polyneuropathy; moreover, few patients suffered from optic neuritis. In few cases, persistent neurosensorial hearing loss has also been described [1, 2, 12].

Most patients have gastrointestinal manifestations: abdominal pain, significant weight loss, chronic gastritis, hepatomegaly, splenomegaly, portal hypertension, bowel perforation or stenosis.

While nephrogenic hypertension is rather common in this condition, in few patients focal glomerulosclerosis and renal amyloidosis have also been described [11]. Lung involvement with necrotising pneumonia (lethal) has been reported in one patient [11].

The blood tests usually reveal an elevation of acute phase reactants (ERS, CRP), low haemoglobin levels and neutrophilic leukocytosis [1, 2]; however in few patients cytopenia (pancytopenia, leucopoenia) has been detected [1, 7, 12]. Auto-antibody are usually negative.

As stated above, a mild immunodeficiency can be observed; some patients present hypogammaglobulinemia that may affect IgM or all Ig subclasses [1, 13]. Of note, despite the low immunoglobulins’ levels, only few cases displayed an increased susceptibility to infections, that was rather severe in exceptional cases [1, 3, 7, 12, 13].

MRI is the most useful tool in the diagnosis of cerebral strokes; in fact CT scan as well as conventional angiography may not detect the smaller lacunar strokes and therefore underestimate the entity of involvement of the CNS [1].

Some patients underwent an angiographic investigation, that revealed the presence of stenosis and/or aneurysms of abdominal artery, particularly mesenteric, celiac, hepatic and renal arteries; the histological analysis, when done, revealed a necrotizing vasculitis [1, 2].

In patients with symptoms suggestive for organ involvement but without pathologic finding in not-invasive radiologic studies, conventional angiography can be of help revealing aneurism and or stenosis in the middle sizes arteries.

Skin biopsy revealed, in most cases, a non-granulomatous, necrotizing vasculitis of small and medium-sized vessels, with the same histopathologic features of polyarteritis nodosa [1, 2, 9].

In few cases the histology was less specific showing a leucocytoclastic vasculitis or a panniculitis.

Polyarteritis nodosa (PAN) is, according to the Chapel Hill classification, a “Necrotizing arteritis of medium or small arteries without glomerulonephritis or vasculitis in arterioles, capillaries, or venules, and not associated with antineutrophil cytoplasmic antibodies (ANCAs)” [22]. It’s gathered in the medium-sized vessels vasculitis, even if it can affect arteries of any size [22].

Being DADA2 a vasculitis with a genetic basis, it has been proposed to group this disease in the vasculitis with a probable cause according to the Chapel Hill classification [11, 22].

Notably, most of the DADA2 patients not only received the histological diagnosis of PAN but also met the EULAR/PRINTO/PRES diagnostic criteria for childhood polyarteritis nodosa (Table 4) [23].

**Table 4:** EULAR/PRINTO/PRES classification criteria for childhood Polyarteritis nodosa (PAN) [23]

	Histopathology or angiographic abnormalities (mandatory) plus one of the five following criteria:	- Histology: necrotising vasculitis in medium or small-sized arteries. - Angiography: aneurysm, stenosis or occlusion of a medium or small sized artery,
EULAR/PRINTO/PRES classification criteria for childhood Polyarteritis nodosa (c-PAN)	1. Skin involvement	Livedo reticularis, skin nodules, superficial ulcers, peripheral tissue necrosis
	2. Myalgia/muscle tenderness	Muscle pain or tenderness
	3. Hypertension	Blood pressure > 95th centile
	4. Peripheral neuropathy	Sensory or motor neuropathy
	5. Renal involvement	Proteinuria, haematuria, impaired function

### Unusual phenotypes

Even if most of the patients with DADA2 have a clinical phenotype consistent with a systemic inflammatory vasculopathy, a recent report has enlighten that the disease may be dominated by clinical manifestations suggestive for an immune-disrective condition, such as cytopenia, lymphadenopathy, hepatosplenomegaly and immunodeficiency with severe viral infections [7]. The two patients described did not present skin involvement and one of them developed a vascular involvement only after bone-marrow transplantation. Of note, the mutations found in these two patients were the same described in patients with a “typical” inflammatory clinical picture.

Similarly a third patient with a lymphoprolipherative clinical picture, resembling Castleman’s syndrome, has been reported by the same group [5].

A more recent clinical series of 9 DADA2 patients with the homozygous R169Q mutation has enlightened that the presence of cytopenia is a common finding of the disease, together with the common inflammatory manifestations [12].

In the two patients carrying homozygous 22q11.1 deletion, encompassing both copies of the IL-17 receptor A (*IL17RA*) and the *CECR1* gene, the clinical phenotype was dominated by muco-cutaneous infections and dermatitis associated to persistent inflammation and, in one patient, vasculitis responding to steroids [14]. Livedo reticularis, stroke and other DADA2 clinical manifestations were not described.

Finally two brothers with a clinical picture consistent with the diagnosis of common variable immunodeficiency (CVID) were found to be affected by DADA2 by whole exome-sequencing; of note only one of them displayed clinical sign and symptoms consistent with a vasculopathy [13].

### Outcome

Being a disease of recent identification, the clinical outcome has not been well investigated. However, from the clinical data by now available is clear that the spectrum of severity of the disease is wide, ranging from patients with neonatal onset and a severe organ involvement to patients with onset in the adulthood and the presence of only skin manifestations (Tables 2 and 3); of note, even between patients carrying the same mutations in *CECR1* gene the clinical picture can be widely different (Tables 2 and 3).

The disease turned out to be lethal in seven out of the 65 patients by now described [1, 2, 6, 13, 14]: in three cases the severity of the visceral involvement was lethal [1, 2], two patients died for respiratory complications following intracranial haemorrhage [6, 13], while one patient developed necrotising pneumonia [1, 11]; finally one of the two patients carrying the homozygous deletion on 22.11.1 chromosome died for septic shock.

### Treatment

DADA2 is a newly recognised condition and the number of patients so far described is limited; for this reason the response to treatment is largely anecdotal and still controversial (Table 5).

**Table 5:** Treatment administrated and clinical response in the described DADA2 patients (1-14)

Therapy	Case report (number of treated patients)	Response to treatment
Steroids (orally or i.v.)	Navon et al. (17)	In 2 cases complete control of the disease with on demand steroidal therapy. In other patients steroid-dependence.
	Zhou et al. (9)	Partial control of diseases manifestations with high doses of corticosteroids
	Van Eyck et al. (2)	Steroid-dependence
	Belot et al. (2)	Steroid-dependence
	Garg et al. (1)	Steroid-dependence
	Van Montfrans et al. (6)	Partial response in 3 patients
	Schepp et al. (1)	Partial response
cyclophosphamide (orally or i.v.)	Navon et al. (9)	Good response in 2 patients.
	Zhou et al. (7)	Not specified
	Belot et al. (2)	Partial response
	Garg et al. (1)	Poor response
	Batu et al. (4)	Poor response
Azathioprine	Navon et al. (7)	No patients with complete response
	Van Eyck et al. (2)	Poor response
	Belot et al. (1)	Good response in association to methotrexate
	Batu et al. (3)	Poor response
	Van Montfrans et al. (5)	Not specified
Methotrexate	Navon et al. (3)	Good response in association with other immunosuppressive and biologics
	Belot et al. (1)	Good response in association with azathioprine
	Batu et al. (3)	Poor response
	Schepp et al. (1)	Partial response
Cyclosporine	Van Eyck et al. (1)	Poor response
Colchicine	Batu et al. (5)	Good response in one patient, none response in 4 patients
Mycophenolate	Zhou et al. (2)	Not specified
	Van Eyck et al. (1)	Poor response
	Belot et al. (1)	Partial response in association with cyclophosphamide
	Batu et al. (2)	Good response in one patient, poor in the other
Sirolimus	Van Eyck et al. (2)	Good response in one patient Poor response in one patient
Tacrolimus	Van Eyck et al. (2)	Good response in one patient Poor response in one patient
I.v. immunoglobulins	Navon et al. (1)	Not specified
	Zhou et al. (5)	Not specified
	Van Eyck et al. (2)	Prophylactic dosage
	Belot et al. (1)	Prophylactic dosage
	Schepp et al. (2)	Prophylactic dosage
Anakinra	Zhou et al. (5)	Not specified
	Garg et al. (1)	Initial partial response than relapse
	Van Montfrans et al. (1)	Good response
Canakinumab	Garg et al. (1)	Initial partial response than relapse
Etanercept	Navon et al. (5)	Complete response in 5 patients Partial response in 1 patient
	Zhou et al. (6)	Not specified
	van Montfrans et al. (3)	Partial response in 1 pateint Complete response in 2 patients
	Batu et al. (3)	Partial response in 2 patients, complete in 1
Adalimumab	Navon et al. (3)	Complete response in 2 patients, exacerbation in 1 patient
Infliximab	Navon et al. (2)	Complete response in 1 patient Partial response in 1 patient
Tocilizumab	Zhou et al. (1)	Not specified
	Van Eyck et al. (1)	Complete response
	Batu et al. (1)	Poor response
Rituximab	Zhou et al. (1)	Poor response
	Belot et al. (1)	Poor response

Being an inflammatory condition, high doses of steroids are usually able to control the clinical manifestations [1, 2, 8, 9, 11, 12]. However, due to the severity of the condition, a steroid-dependence is often described. None of the most common immunosuppressive drugs (cyclophosphamide, azathioprine, methotrexate) was effective [1, 2, 6, 8, 11, 13].

Navon et al. reported ten patients treated with anti-TNF drugs (etanercept, adalimumab, infliximab) with complete response in 8, even after the failure of immunosuppressive therapies [2]; good results with anti-TNF agents were also reported in other small series [3, 11, 12]. By now, the reason why this drug is effective is still unclear.

According to the report of Zhou et al., neither immunosuppressive nor biologic drugs were able to completely control the disease manifestations in all treated patients; the enzymatic substitutive treatment (fresh frozen plasma or recombinant enzyme) was postulated to be of help. This approach was tempted in two patients reported by Batu et al. with a transient good response in one and a not-satisfactory response in the other [11].

A possible role of hematopoietic stem cell transplantation (HSCT) has been postulated to be effective by Zhou et al. and Navon et al., being able to provide ADA2 producing monocytes and therefore to normalize the plasmatic levels of the enzyme [1, 2]. This therapeutic strategy, performed in one of the two patients reported by Van Eyck et al. [7] and in a patient reported by the NIH group [3], was able to normalize the plasmatic levels of ADA2 and to control the disease manifestations [3, 7]; early complications occurred in one of them. More recently two additive patients who displayed a complete response to HSCT have been described [12].

Van Eyck et al. conclude that HSCT should be suggested only for those patients with a severe disease, since DADA2 patients present an increased risk of HSCT-related complications due to the persistent inflammation and the compromised endothelial integrity [7]. Of note, the other patient described in this paper displayed a complete response to treatment with sirolimus; the authors assume that this drug may be of help in the control of the clinical manifestations related to ADA2-deficiency, being able to reduce the M1 macrophage differentiation and the production of IL-6 [7].

## Conclusion

In conclusion DADA2 is a genetic condition mainly characterized by an inflammatory vasculopathy resembling polyarteritis nodosa (PAN). From the clinical data so far available, the age at onset, the disease manifestations and severity are widely variable. Further clinical studies are therefore needed in order to better understand the phenotypic viability of this condition and the genotype-phenotype correlation.

In light of the data by now available, we consider the genetic analysis of *CECR1* gene suggested in the following clinical pictures: patients with an inflammatory vasculopathy with early onset in infancy, patients with a diagnosis of PAN or cPAN with early onset and/or severe organ involvement (above all stroke), especially in case of a positive family history or consanguinity/endogamy in the parents. Moreover DADA2 should be ruled out in patients with an immune-disreactive condition without an underlying diagnosis, especially in presence of signs or symptoms of vasculitis.

Finally, a better enlightenment of the pathogenetic mechanisms of the disease is needed; these data will be of help also in the identification of an effective treatment.
